# Increased risk of allergic rhinitis among children delivered by cesarean section: a cross-sectional study nested in a birth cohort

**DOI:** 10.1186/s12887-016-0594-x

**Published:** 2016-04-27

**Authors:** Heli Vieira Brandão, Graciete Oliveira Vieira, Tatiana de Oliveira Vieira, Paulo Augusto Camargos, Carlos Antonio de Souza Teles, Armênio Costa Guimarães, Alvaro Augusto Cruz, Constança Margarida Sampaio Cruz

**Affiliations:** Doctorate student of Escola Bahiana de Medicina e Saúde Pública, Salvador, Brazil; Full Professor of Pediatrics, State University of Feira de Santana, Feira de Santana, Brazil; Instructor of Pediatrics, State University of Feira de Santana, Feira de Santana, Brazil; Full Professor of Pediatrics, Federal University of Minas Gerais, Belo Horizonte, Brazil; Associate Professor State University of Feira de Santana, Feira de Santana, Brazil; Full Professor of Escola Bahiana de Medicina e Saúde Pública, Salvador, Brazil; Head of the Center of Excellence in Asthma of the Federal University of Bahia, Salvador, Brazil; Adjunct Professor of Escola Bahiana de Medicina and Saúde Pública. Coordinator of Multidisciplinary Research of Hospital Santo Antonio, Obras Sociais Irmã Dulce, Salvador, Brazil; Rua Marechal Castelo Branco 597, Capuchinhos, Feira de Santana, Bahia CEP: 44076-020 Brazil

**Keywords:** Asthma, Allergic rhinitis, Cesarean, Children

## Abstract

**Background:**

Few studies have evaluated the association between delivery by cesarean section (CS) and asthma, allergic rhinitis and chronic rhinitis and whether this association is different in children with and without a family history of asthma. This study aims to investigate whether children born by CS have a higher chance to develop asthma, allergic rhinitis and chronic rhinitis and to evaluate the influence of parental history of asthma on these associations.

**Methods:**

This is a cross-sectional study of 672 children nested in a birth cohort evaluated at 6-years of age. Asthma and chronic/allergic rhinitis were identified by means of the mother’s responses to the ISAAC questionnaire. The association between CS, asthma, chronic rhinitis and allergic rhinitis was evaluated by multivariable logistic regression. The evidence of effect modification of parental history of asthma on the association CS and outcomes was examined by introducing interactions terms in the logistic regression models adjusting for confounders.

**Results:**

Asthma was not associated with birth by CS irrespective of parental history of asthma (odds ratio (OR) 1.03; 95 % CI 0.61–1.74). Chronic rhinitis and allergic rhinitis were both significantly associated with birth by CS but only in the subgroup of children with by parental history of asthma (OR 1.56; 95 % CI 1.04–2.34) and (OR 1.60; 95 % CI 1.01–2.55) respectively, after adjustment for confounders. The parental history of asthma was a effect modifier in the association between CS, chronic rhinitis and allergic rhinitis (*p* for effect modification = 0.10 and 0.02, respectively).

**Conclusion:**

CS increases the risk of chronic rhinitis and allergic rhinitis in children at 6 years of age with parental history of asthma. Health professionals must be alerted with regard to the increased risk of allergic rhinitis and made aware this is another reason to avoid unnecessary CS.

## Background

The prevalence of asthma and chronic rhinitis are high in Brazil [1, 2]. The average prevalence rate of symptoms of asthma in the age range 6 and 7 years is 24 % and for chronic rhinitis, 25.7 % [3]. In Feira de Santana, State of Bahia, the prevalence of symptoms of chronic rhinitis and allergic rhinitis was 33 and 17 %, respectively, in the year of the 2003, among the highest in Brazil [2, 3]. The prevalence rate of asthma physician-diagnosed among adolescents is 12.4 and 6.4 % in children 6 to 7 years old [3, 4].

The frequency of birth by cesarean section (CS) is growing fast in the Brazilian public and private health services. The rate of CS among all births was 33.8 % in 2004 and increased to 45.9 % in 2008 [5], being considerated the highest in the world. The World Health Organization recommends a rate of CS of less than 15 %. However, in the majority of countries the actual rate exceeds this recommendation [6].

Several studies have demonstrated an association between symptoms of asthma and allergic rhinitis with CS, but there are controversial results reports [7]. Some cohort studies demonstrated an association between CS and asthma in children, with odds ratio (OR) ranging from 1.09 to 1.82 [8, 9]; whereas other studies have found no association [10, 11]. In Brazil, the study of Menezes et al. evaluated the association between CS and asthma in children in two age ranges and did not find a significant association [12]. A meta-analysis has favored a positive association with an estimate increased risk of 20 % for having asthma among children born by CS (Thavagnanam et al., 2008) [13].

There are a few studies that have evaluated the association between allergic rhinitis, asthma and birth by CS in children with genetic predisposition, and in those that have demonstrated a positive association, the OR ranged between 1.37 and 1.80 [14, 15]. Nevertheless, a meta-analysis have not demonstrated a significant increase in the risk of allergic rhinitis in children born by CS (Bager et al., 2003) [16].

The aim of the present study was to investigate whether children born by CS have a higher chance of developing asthma and allergic rhinitis at 6 years of age, looking for other potential risk factors in a birth cohort and evaluating family history of asthma as a possible effect modifier.

## Methods

### Study design

This is a cross-sectional study nested in a birth cohort.

### Study population

A total of 684 mother-child dyads provided the basis for this study, as they were followed-up from birth and the children could be reviewed at 6 years of age. The births occurred in all public and private hospitals in the municipal district in the period from April 2004 to March 2005 in Feira de Santana, a city (608,000 inhabitants) located in Bahia, Northeast in Brazil.

The study was conducted in all ten hospitals of the city (namely, Emec, Cleriston Andrade, Inácia Pinto dos Santos, D. Pedro de Alcântara, São Mateus, Mater day, Santa Cecília, Stela Gomes, Unimed and Casa de Saúde Santana) in the period of 1 year, with the inclusion of all the children that were born in the period of two consecutive months in each hospital (*n* = 1.344).

The sample size calculation used for this study was estimated by the program OpenEpi (Open Source Epidemiologic Statistics for Public Health, version 2.3.1) adopting the following parameters: interval of confidence of 95 %, statistical power of 80 %, statistical significance α = 0.05, estimated risk of odds ratio equal to 2 for association of CS with asthma and a difference in prevalence of 7.9 %. The sample size estimated was 582 children, with 291 in each group (CS and vaginal delivery). However, considering the possibility of losses we included in the study all 684 children from the cohort we were able to find. There was no difference in baseline characteristics (gender of newborn, birth weight, gestational age) between participants and children lost to follow up. The losses were probably related to the frequent mobility of the low-income population of this region.

The following non-inclusion criteria were used upon enrollment for the study: children born of mothers who presented complications during gestation (eclampsia, placenta previa) or in the neonatal period (perinatal hyoxia, newborn respiratory distress syndrome). All women included in the study provided written informed consent.

### Exposure variable

The exposure of interest was delivery by CS (including elective and emergency CS) and vaginal delivery.

### Definitions of asthma, chronic rhinitis and allergic rhinitis

The dependent variables analyzed were active asthma/severe asthma and chronic /allergic rhinitis. The criteria adopted in this study for the definition of asthma, chronic rhinitis and allergic rhinitis were same adopted from the ISAAC study questionnaires [1]. A standard questionnaire was applied to the mothers at home visits when the child was 6 years old.

For the identification of active and severe asthma the following questions were considered: wheezing in the last 12 months (to identify active asthma symptoms) and episodes of wheezing that would prevent the child from speaking two or more words between one breath and the other (severe asthma episode). For the identification of rhinitis the following questions were considered: nasal symptoms of rhinorrhea (runny nose), obstruction, itching and sneezing in the last 12 months, without a flu or cold (to identify chronic rhinitis) and symptoms nasal accompanied by itchy or watery eyes in the last 12 months (as the indicator of allergic rhinitis).

### Covariates

The following factors that could influence on the association of asthma with CS were evaluated: mother's age (<20 years, ≥ 20 years); family income (up to 2 minimum wages, > 2 minimum wages); mother's educational level (up to 8 years of schooling, > 8 years of schooling); maternal smoking during gestation (yes, no); gestational age (<37 weeks, ≥37 weeks); gender of the newborn (male, female); birth weight (<2.500 kg, ≥2.500 kg); breastfeeding up to 4 month (yes, no); number of persons who sleep in the same room with the child (<4 people, ≥4 people); use of nursery before 2 years of age (yes, no); history of a clinical condition compatible with acute viral bronchiolitis in the 1st year of life (yes, no) and physician-diagnosed pneumonia ever (yes, no); type of hospital (private, public). Parental history of asthma was stratified into two groups as follows, parental history of asthma and no parental history of asthma.

Information related to the perinatal period was obtained in the first 72 h after birth, by means of a questionnaire applied to the mothers by trained interviewers, and confirmed by records on medical charts. Data related to the postnatal period were obtained in scheduled home visits during the period of follow-up of the children in cohort.

### Statistical analysis

The samples characteristics were compared by the chi-square test and Student *t* test, according to the type of delivery. The prevalence estimate according to type of delivery for active/severe asthma, allergic/chronic rhinitis were calculated by dividing the number of positive responses to the given question in the ISSAC questionnaire by the total number of participants expressed as percentages.

The associations between CS, asthma, allergic and chronic rhinitis were evaluated by using a multivariate logistic regression test adjusting for potential confounders (family income, educational level; smoking during gestation; gestational age; birth weight; breastfeeding up to 4th month; number of persons who sleep in the same room as the child; use of nursery before age 2 years; physician-diagnosed pneumonia ever). A p-value of <0.05 was considered to be statistically significant. The effect modification of parental history of asthma on the association between CS, asthma, chronic rhinitis and allergic rhinitis was checked by including an interaction term between parental history of asthma and CS in the multivariable logistic regression models. Evidence for effect modification was assessed by the Likelihood Ratio Test (comparing the goodness of fit of models with and without the interaction term).

The data were analyzed with the Program SPSS version 4 (Statistical Package of Social Sciences) and by STATA 13 (StataCorp LP, College Station, Texas, USA).

### Ethical aspects

The protocol and respective informed consent for the project were approved by the Research Ethics Committee of Feira de Santana State University (Report No. 04775012.8.0000.0053). Furthermore, all ten participating hospitals only allowed data collection in their respective units upon submission of approval by the Committee of Research Ethics. All women included in the study provided written informed consent.

## Results

In this study 672 mother-child dyads (98.2 %) of the 684 being currently followed-up in the cohort were included in analysis. The 12 remaining dyads were not identified because of recent change of their home addresses.

The rate of CS was 48.3 % and there was no significant differences between the two groups divided by type of birth in relation to birth weight, gender, gestational age, pneumonia ever, bronchiolitis in the 1st year of life and maternal smoking during gestation. However, different mother’s ages at the time of birth, family income, mother’s schooling up to 8 years of study, number of people who sleep in the room with the child, attendance of a nursery to the age of 2 years and institution where the birth took place (private/public) were different when comparing children’s birth by CS versus vaginal delivery (Table 1).

**Table 1:** Characteristics of mothers and children delivered by cesarean and vaginal delivery

Variables	Total (*n* = 672) *N* (%)	Cesarean delivery (*n* = 325) *N* (%)	Vaginal delivery (*n* = 347) *N* (%)	*P* ^**^
Current age of the child in months (X ± SD) ^a^	72.66 (5.32)	72.97 (5.18)	72.38 (5.43)	0.149
Age of the mother at the time of birth				
< 20 years	104 (15.5)	33 (10.2)	71 (20.5)	<0.001
≥ 20 years	568 (84.5)	292 (89.8)	276 (79.5)	–
Gender of newborn				
Male	336 (50)	156 (48)	180 (51.9)	0.316
Female	336 (50)	169 (52)	167 (48.1)	–
Weight at birth				
< 2.500 kg	30 (4.5)	312 (96)	330 (95.1)	0.570
≥ 2.500 kg	642 (95.5)	13 (4)	17 (4.9)	–
Gestational age				
<37 weeks	28 (4.2)	24 (7,4)	19 (5.5)	0.079
≥37 weeks	644 (95.7)	301 (92.6)	328 (94.5)	–
Family income				
Up to 2 minimum wages	477 (71)	153 (47.1)	42 (12.1)	<0.001
> 2 minimum wages	195 (29)	172 (52.9)	305 (87.9)	–
Mother’s schooling level				
Up to 8 years of schooling	202 (30.1)	265 (81.5)	205 (59.1)	<0.001
> 8 years of schooling	470 (69.9)	60 (18.5)	142 (40.9)	–
Exclusive breastfeeding up to the 4^th^ month				
Yes	137 (20.4)	76 (23.4)	61 (17.6)	0.062
No	535 (79.6)	249 (76.6)	286 (82.4)	–
Smoking during pregnancy				
Yes	20 (3)	10 (3.1)	10 (2.9)	0.882
No	652 (97)	315 (96.9)	337 (97.1)	–
Number of persons who sleep in the room with the child				
<4 people	641 (95.1)	316 (97.2)	325 (93.7)	0.027
≥4 people	31 (4.6)	9 (2.8)	22 (6.3)	–
Frequency at nursery to the age of 2 years				
Yes	104 (15.5)	74 (22.8)	30 (8.6)	<0.001
No	568 (84.5)	251 (77.2)	317 (91.4)	–
Pneumonia ever in life				
Yes	41 (6.1)	17 (5.2)	24 (6.9)	0.362
No	631 (93.9)	308 (94.8)	323 (93.1)	–
Bronchiolitis in the 1st year				
Yes	65 (9.7)	27 (8.3)	38 (11)	0.247
No	607 (90.2)	298 (91.7)	309 (89)	–
Maternities				
Private	199 (29.6)	154 (47.4)	45 (13)	<0.001
Public	473 (70.4)	171 (52.6)	302 (87)	–

^**^ Chi-square Test

^a^ Student’s Test

The overall prevalence of asthma symptoms was 13.8 %, being 14.8 % and 13 % among subjects born by CS and vaginal delivery respectively. There was no statistically significant difference in the prevalence of symptoms of active asthma or severe asthma episodes between the children born by CS or vaginal delivery.

The prevalence rate of symptoms related to chronic rhinitis was 36.9 %, and of allergic rhinitis, 23.5 %; The prevalence of symptoms of chronic and allergic rhinitis was higher in children born by CS when compared with those born by vaginal delivery (Table 2).

**Table 2:** Association between type of delivery, asthma and rhinitis

Variables	Frequency (%)		OR (95 % CI); *p* value	
	Caesarean Delivery (325)	Vaginal Delivery (*n* = 347)	Unadjusted	Adjusted^e^
Asthma				
Active asthma ^a^	48 (14.8 %)	45 (13 %)	1.16 (0.75–1.80); 0.499	1.03 (0.61–1.74); 0.883
Severe asthma^c^	27 (8.3 %)	22 (6.3 %)	1,31 (0.76–2.25); 0.327	1.15 (0.47–2.84); 0.746
Rhinitis				
Chronic rhinitis ^b^	138 (42.5 %)	110 (31.7 %)	1.59 (1.16–2.18); 0.004	2.47 (1.56–3.89); <0.001
Allergic rhinitis^d^	92 (28.4 %)	66 (19 %)	1.68 (1.17–2.42); 0.004	1.86 (1.18–2.93); 0.007

^a^ Wheezing in the past 12 months

^b^ Symptoms of rhinitis in the past 12 months

^c^ Intense sibilance episodes, restricting speech (two successive words) in the past 12 months

^d^ Rhinoconjunctivitis symptoms in the past 12 months

^e^Adjusted for gestational age, weight at birth, breastfeeding up to the 4th month, mother’s smoking during pregnancy, family income, mother’s schooling level, parity, number of persons who sleep in the room with the child, attendance to nursery to the age of 2 years, pneumonia ever

Active and severe asthma symptoms were not associated with delivery by CS, even after the adjustment for the following covariates: gestational age, birth weight, breastfeeding up to the 4th month, mother’s smoking during gestation, family income, mother's educational level, number of siblings, number of persons sleeping in the same room as the child, attendance to nursery before the age of 2 years and physician-diagnosed pneumonia ever. The frequency of CS section and vaginal delivery in children with active asthma was 51.6 % versus 48.4 % respectively (adjusted OR 1.16; 95%IC 0.75–1.80). Chronic and allergic rhinitis were associated with delivery by CS in the bivariate analysis as well as in the multivariate logistic regression adjusted for the same covariates as for asthma (Table 2).

Table 3 demonstrates the frequency of asthma in children delivered by CS and vaginal route with and without parental history of asthma. In children born by CS, the parental history of asthma was associated with asthma (*OR* = 1,97; 95 % CI 1.04–3.74) whereas in children born by vaginal delivery, the parental history of asthma was also associated with asthma in the child (*OR* = 2.05; 95 % CI 1.07–3.94). There was also an association between birth by CS and symptoms of chronic rhinitis and allergic rhinitis only in children with a parental history of asthma, after adjusting for all covariates, as demonstrated in Table 3.

**Table 3:** Association between type of delivery, asthma and rhinitis according to parental history of asthma (*n* = 672)

Variables		No parental history of asthma (*n* = 496)			Parental history of asthma (*n* = 176)		Effect modification
	*N* (%)	OR (95 % CI) Unadjusted	OR (95 % CI) Adjusted	*N* (%)	OR (95 % CI) Unadjusted	OR (95 % CI) ^a^Adjusted	*p*-value^**^
Active asthma							
Cesarean delivery	29 (12.2 %)	1.18 (0.68–2.07)	1.31 (0.71–2.41)	19 (20.4 %)	1.07 (0.72–1.40)	1.14 (0.43–2.99)	0.78
Vaginal delivery	27 (10.5 %)	–	–	18 (19,4 %)	–	–	–
Severe asthma							
Cesarean delivery	18 (7.6 %)	1.77 (0.85–3.67)	2.14 (0.90–5.06)	11 (12.4 %)	1.33 (0.82–2.18)	2.20 (0.96–5.08)	0.31
Vaginal delivery	11 (4.3 %)	–	–	9 (10.3 %)	–	–	–
Chronic rhinitis							
Cesarean delivery	99 (41.6 %)	1.84 (1.26–2.64)	1.43 (0.94–2.18)	39 (44.8 %)	1.09 (0.60–1.97)	1.56 (1.04–2.34)	0.10
Vaginal delivery	72 (27.9 %)	–	–	38 (42.7 %)	–	–	–
Allergic rhinitis							
Cesarean delivery	63 (26.6 %)	1.71 (1.11–2.63)	1.46 (0.91–2.36)	29 (33.3 %)	1.61 (0.63–3.13)	1.60 (1.01–2.55)	0.02
Vaginal delivery	45 (17.4 %)	–	–	21 (23.6 %)	–	–	–

^**^ *P*-value for effect modification by parental history of asthma in multivariable models

^a^ Adjusted for gestational age, weight at birth, breastfeeding up to the 4^th^ month, mother’s smoking during pregnancy, family income, mother’s schooling level, parity, number of persons who sleep in the room with the child, attendance to nursery to the age of 2 years, pneumonia ever

Therefore the effect modifier of parental history of asthma was demonstrated in children born by CS, as they were significantly more likely to develop allergic rhinitis and chronic rhinitis, when exposed to the two variables combined (p value for Likelihood Ratio Test for effect modification = 0.02 and 0.10, respectively) (Table 3).

## Discussion

This study demonstrated a positive association between CS, chronic rhinitis and allergic rhinitis, after adjusting for potential confounders, but the association was significant only in children at higher risk of chronic rhinitis and allergic rhinitis as indicated by parental history of asthma. CS was not significantly associated with symptoms of asthma at age of 6 years in our study population. A cross-sectional study by Kolokrotoni et al. [17] found a positive association between CS, asthma and atopic sensitization in children aged 8–9 years. When children were stratified by family history of allergy, the association between CS and atopic sensitization was more pronounced but this was not the case for asthma outcomes. While more than a two-fold increase in the odds of being atopic was observed in children with a family history of allergy if born by CS, no association was observed in children without a family history of allergies.

The diagnosis criteria of active asthma considered in the present study was based on symptoms of the disease in the 12 months before the application of the ISAAC questionnaire, ruling out the cases of transient wheezing at an earlier age.

The possibility of selection bias doesn’t seem to be relevant in the present study, because children born by CS did not differ significantly from those born by vaginal delivery with regard to the main confounding variables (gestational age, birth weight, time of exclusive breastfeeding and mother smoking during gestation). Moreover, there were not differences between the proportions of exclusions due to complications during gestation or related to birth, such as differences in proportions of newborns who presented perinatal respiratory complications. The absence of association between CS and asthma may be explained because the majority of cases of symptoms of asthma related symptoms reported in urban centers in Brazil have not been attributed to atopy, as demonstrated in a study in children conducted in Salvador-Bahia, just 100 km from Feira de Santana, where the present study was conducted, by Cunha et al. [18].

Previous studies that assessed the association between CS and asthma presented different results. Roduit et al. [19], in a prospective cohort study of 2.917 children, found a significant association between delivery by CS and asthma (*OR* = 1.77) and the risk was increased in the presence of parental history of atopy. In a prospective cohort study of 12,367 children, Maitra et al. [20] found no association between delivery by CS and asthma (*OR* = 1.14; 95%CI 0.9–1.4). In two populations included in a cohort study conducted in Southern of Brazil by Menezes et al. [12], no significant association was found between the type of birth and dyspnea at 4 years of age (*OR* = 0.96), nor from 4 to 11 years (*OR* = 1.18) neither at 15 years (*OR* = 1.02). A meta-analysis conducted by Bager et al. [16], found a significant increase in the risk for asthma in children delivered by CS with (*OR* = 1.18;95%CI1.05–1.32) and risk for allergic rhinitis with (*OR* = 1.24;95%CI 1.08–1.43) [17]. In another meta-analysis, Thavagnanam et al. also found an increased risk for asthma in children delivered by CS (*OR* = 1.22; 95%CI 1.14–1.29) [13]. However, in these analyses there was high heterogeneity among the studies. They differed in many issues such as definition of asthma, age of the children when the diagnosis of asthma was made, in the study design, evaluation of environmental factors, time duration of exclusive breastfeeding and the use previous of BCG vaccination [21, 22].

The significant association between delivery by CS, chronic and allergic rhinitis observed in the present study was also identified in the studies carried out by Pistiner e et al. (*OR* = 1.80); Renz-Polster e et al. (*OR* = 1.37); Salam e et al. (*OR* = 1.57) [8, 14, 15]. Other studies found no association between delivery by CS and allergic rhinitis: Park e et al. (*OR* = 1.14); Xu e et al. (*OR* = 1.28); Mallen e et al. (*OR* = 2.82) [10, 23, 24]. Parental history of atopy is considered an important risk factor for the development of asthma and atopic diseases [25], and is a potential effect modifier in the evaluation of the association between delivery by CS, asthma, allergic rhinitis and atopic sensitization [14, 15, 17]. In our study, CS was associated allergic rhinitis and chronic rhinitis only in children with parental history of asthma, which suggests that the association between CS and allergic rhinitis and CS and chronic rhinitis could be dependent on the genetic predisposition. Our finding is consistent with the results of study of Pistiner e et al. which found increased risk of allergic rhinitis among children with a parental history of atopy but did not find any evidence of a positive association between CS and asthma in children with parental history of atopy.

The controversial reports on the association between allergic rhinitis and delivery by CS may be due to the different diagnostic criteria considered for the definition of allergic rhinitis, or the presence of confounding factors, such as maternal smoking during gestation, mother's history of asthma, and primiparity [26]. In the present study, the inclusion of the main confounding variables in the multivariate analysis of the association between delivery by CS and allergic rhinitis might have contributed to a greater discrimination of the findings.

In the present study the birth rate by CS was higher in children born in private hospital, reproducing the current situation of the type delivery that occurs in Brazil [5]. Therefore differences between children born by CS and vaginal delivery may be related to socioeconomic differences between children born in public health services versus private hospitals.

The possible biological mechanism that may explain the direct association between delivery by CS and allergic rhinitis could be related to the hygiene hypothesis: the characteristics of the intestinal microbiota in children born by CS is different from that found among children born by vaginal delivery [27]. The intestinal microflora of children born by vaginal delivery is composed of bacteria from the vaginal flora by exposure while in the birth canal, whereas the intestinal biome of children born by CS is composed of bacteria from colonization of the skin and those related to the hospital environment [28]. According to the hygiene hypothesis, there would be a predominance of the immunologic response of the Th2 type in children born by CS, due to the lack of physiological modulation of the immune system conferred by the natural biome. That condition entails the later development of atopic asthma, allergic rhinitis and other atopic diseases [29]. Children born by CS present elevated levels of IL-13 and interferon γ in the umbilical cord and lower levels of IL-10, a regulatory cytokine [30].

The limitations of our study could be related to the lack of a multilevel approach analysis given the 10 institutions where participants were selection; the study design not robust enough to infer causation, as it did not allow the collection of biomarkers of atopy (skin prick tests or specific IgE), which could have reinforced the causality association by bringing in mechanistic evidence; we had no stool cultures to examine the type of intestinal microflora present in the children born by different methods, which could also bring further evidence to the theoretical explanation; possible misclassification due to self-reported of outcomes, as to parental history of asthma related to mothers; lack of classification of the CS type (emergency and elective), considering that babies born through emergency CS might be at less risk of allergies due to the exposure of the children to vaginal flora during labor. Despite these limitations, the results were similar to previous ones carried out in different settings and with different populations, and provide further evidence to fill the gap of knowledge on the association between, chronic and allergic rhinitis in children with parental history of asthma.

Our results indicate that elective CS increases significantly the risk of developing chronic rhinitis and allergic rhinitis, chronic diseases that imply in recurring discomfort and impaired quality of life among children with a family predisposition for asthma.

## Conclusions

In conclusion, birth by CS was associated with both chronic rhinitis and allergic rhinitis in children with parental history of asthma, in this cross sectional study nested in a birth cohort.
